# Consensus-based recommendations of Australian podiatrists for the prescription of foot orthoses for symptomatic flexible pes planus in adults

**DOI:** 10.1186/s13047-014-0049-2

**Published:** 2014-11-25

**Authors:** Helen A Banwell, Shylie Mackintosh, Dominic Thewlis, Karl B Landorf

**Affiliations:** International Centre for Allied Health Evidence (iCAHE), School of Health Sciences, University of South Australia, Adelaide, South Australia 5001 Australia; Biomechanics and Neuromotor Laboratory, School of Health Sciences, University of South Australia, Adelaide, South Australia 5001 Australia; Sansom Institute for Health Research, University of South Australia, Adelaide, South Australia 5001 Australia; Department of Podiatry and Lower Extremity and Gait Studies Program, Faculty of Health Sciences, La Trobe University, Victoria, 3083 Australia

**Keywords:** Foot orthoses, Delphi study, Consensus, Pes planus, FootPROP

## Abstract

**Background:**

Foot orthoses are commonly used for symptomatic flexible pes planus in adults. However, there are no clinical guidelines for the prescription of customised foot orthoses that are specific to this population. The aim of this study was to investigate prescription habits of Australian podiatrists for customised foot orthoses for symptomatic flexible pes planus in adults and to develop consensus-based practice recommendations for the prescription of these foot orthoses.

**Methods:**

A four round Delphi survey was undertaken with 24 podiatric experts to establish current use and rationale for individual prescription variables of customised foot orthoses for symptomatic flexible pes planus in adults. Round one determined prescription use (consensus) and rounds two, three and four determined the rationale for use (agreement) of prescription variables across the rearfoot, midfoot, forefoot, as well as accommodation and materials used. For consensus and agreement to be accepted, 70% of the respondents were required to use or agree on the rationale for use of individual prescription variables.

**Results:**

Consensus was reached in round one for two variables, choice of shell material (polyolefin) and when to prescribe a forefoot post balanced to perpendicular. In rounds two, three and four, agreement was reached for 52 statements related to the rationale for use of individual prescription variables, including when to prescribe: an inverted cast pour [heel in an inverted position], an inverted rearfoot post, a medial heel (Kirby) skive, minimal/maximum arch fill, a medial flange, a forefoot post and common orthotic accommodations.

**Conclusion:**

This study found consensus or agreement for the use of several prescription variables for customised foot orthoses for symptomatic flexible pes planus in adults. The findings were used to develop the Foot orthosis Prescription Recommendations for symptOmatic flexible Pes planus in adults (FootPROP) proforma, to guide clinicians and researchers in the prescription of customised foot orthoses for this population.

**Electronic supplementary material:**

The online version of this article (doi:10.1186/s13047-014-0049-2) contains supplementary material, which is available to authorized users.

## Background

Pes planus (also known as flat or low-arched foot) has been estimated to affect up to 23% of the adult population [1-5]. The condition is classified as rigid or flexible. Rigid pes planus is defined as ‘a congenital, rigid or spastic deformity of the foot’, and flexible pes planus as ‘an acquired joint disorder resulting in a valgus foot deformity’ [6]. Flexible pes planus may be asymptomatic with little justification for intervention [7] or symptomatic where pain and/or functional limitations are present [8-10]. Accordingly, symptomatic flexible pes planus is a common reason for people seeking intervention [11].

Foot orthoses (FOs) are the most commonly cited intervention for flexible pes planus [12-14]. FOs are in-shoe devices that aim to alleviate symptoms, improve function and prevent injury [13-16]. There are no universally accepted classifications for FOs, although they are often broadly categorised as prefabricated or customised [17-19]. A customised FO is tailored for the individual based on a three-dimensional impression or image of the plantar foot with adjustments made to influence foot alignment and position [20]. Individual customisation of FOs potentially offers selective and targeted intervention [21]. However, guidelines for prescribing customised FOs for specific foot types, including symptomatic flexible pes planus, are yet to be established, and the literature that is available offers minimal direction or consistency [22-24].

As a consequence, researchers and clinicians investigating the effect of FOs are left to choose the type or prescription with little guidance. A recent systematic review on the use of FOs for pes planus found that the majority of studies standardised the type or prescription approach across all participants [25]. However, a common criticism of customised FOs used in clinical trials is that they are not appropriately prescribed; the underlying concern being that a standardised prescription may not address individual foot morphology and function in the same manner as an individual prescription [21].

With minimal evidence to base prescriptions of FOs for flexible pes planus in adults on, seeking expert podiatry opinion is appropriate as a first step in developing prescription guidelines. Consensus methods are considered a useful method of dealing with conflicting evidence in the absence of guidelines [26-28]. Therefore, the aim of this study was to investigate prescription habits of Australian podiatrists for customised FOs for symptomatic flexible pes planus in adults and to develop consensus-based recommendations for the prescription of these FOs.

## Methods

**Figure 1:**
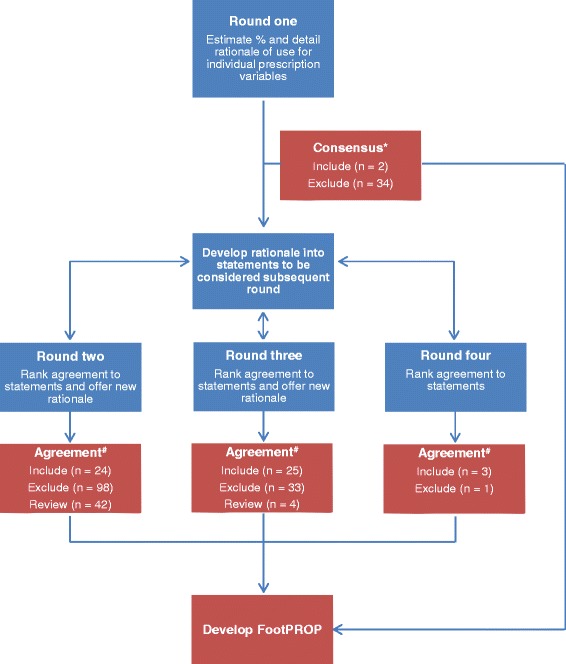
**The Delphi survey four round process and results.** *Data were considered to have reached consensus if the estimated percent of median use for an individual prescription variable, or the preference for a specified type of FO, was 70% or more. Data not reaching 70% were excluded. ^#^Data were considered to have reached agreement if 70% or more of respondents indicated they agreed or strongly agreed with the statement on a five point Likert scale. Data not reaching 50% agreement were excluded. Data receiving 50 – 69% agreement were reviewed in subsequent rounds. All data not reaching 70% agreement at the conclusion of the Delphi were excluded.

This study was a four round Delphi survey. A modified Delphi survey was used where participants’ opinion was sought in round one, and then responses collated and analysed for existing consensus [29]. Responses not reaching consensus were returned to participants for consideration, comment and ranking for levels of agreement in subsequent rounds (Figure 1). The study was approved by the University of South Australia’s Human Research Ethics Committee (protocol number 26682). All participants provided written informed consent prior to completing the survey.

### Participants

Twenty four expert podiatrists were recruited as participants for the Delphi survey panel. An expert podiatrist was defined as a registered podiatrist who had been practicing for 10 years or more and satisfied at least one of the following inclusion criteria: held an academic position teaching podiatric biomechanical theory or clinical practice within an Australian podiatry program; held a clinical position where the practice was focused on biomechanical assessment and intervention; or had published research on lower limb biomechanics or FOs within the past five years.

Forty-five potential participants were identified via discussions with university leaders of podiatry programs in Australia, as well as current and recent chairs of the Podiatry Board of Australia and Australian Podiatry Associations within each state of Australia. Participants were randomly selected from this list, which was weighted by population of state or territory (Additional file 1), and invited to participate until 24 experts were enrolled. This number of participants captured a broad representation of experts in the podiatry profession in Australia whilst remaining manageable in terms of panel size. Participants completed a preliminary survey of participant characteristics to ensure eligibility for the study. Participants were asked to keep their involvement confidential and intra-panel communication was anonymous through-out the survey.

### Survey format

The Delphi survey was implemented using SurveyMonkey® (SurveyMonkey Inc, Palo Alto, California, USA). Prior to the commencement of the main study the survey was piloted in two stages with seven podiatrists who were not participants, to refine the format and question design.

Round one of the main study included questions relating to common prescription variables for FOs across four sections: rearfoot, midfoot, forefoot, as well as accommodations and materials (Additional file 2). It was considered that terminology related to prescription variables and orthosis manufacturing may vary between the states and territories of Australia, therefore ‘traditional’ terms were adopted for the length of the study (e.g. the prescription of ‘cast pour’ was surveyed in preference to newer milling procedures) (Table 1).

**Table 1:** **An overview of the individual prescription variables**

**Prescription variable**	**Method of manufacture**
Cast Pour	Negative cast is held in the prescribed position, based on a bisection of the posterior heel relative to the supporting surface, while liquefied plaster is poured into negative cast
Inverted	Indicates that the negative cast, when poured, is held in an inverted position relative to the heel bisection
Neutral	Indicates that the negative cast, when poured, is held in a vertical position relative to the heel bisection
Everted	Indicates that the negative cast, when poured, is held in an everted position relative to the heel bisection
Medial heel (Kirby) skive	Indicates a small amount of plaster is skived away from the medial heel of the positive cast (skive is generally angled 15 degrees varus/inverted to the plane of the plantar surface of the forefoot post)
Rearfoot post	An addition, typically fashioned from a heat mouldable material, that is applied to the final orthosis to stabilise the heel in a vertical position or angle it in the frontal plane (also known as a heel stabiliser)
No post	No external rearfoot post
Extrinsic	An external heel post that stabilises the orthosis in a vertical position
Extrinsic (inverted)	An external heel post that tilts the orthosis into an inverted position
Extrinsic (everted)	An external heel post that tilts the orthoses into an everted position
Extrinsic (with motion)	An external heel post that has a bi-planar grind on the plantar aspect
Arch fill	The plaster expansion applied to the medial longitudinal arch area of the positive cast
Minimal	A decreased plaster expansion
Standard	A standard plaster expansion
Maximum	An increased plaster expansion
Flange	A midfoot extension of the final orthosis border, typically prescribed in conjunction with a deep and distally extended heel cup
Medial	A superomedial extension
Lateral	A superolateral extension
Forefoot post	A corrective reference platform applied to the medial and/or lateral forefoot
Balanced to perpendicular	The reference platform applied to the plantar forefoot to hold the forefoot alignment as parallel to the supporting surface and perpendicular to the rearfoot
Intrinsic	The reference platform is applied to the positive cast
Extrinsic	The platform is applied to the shell of the final orthosis
No post	No reference platform applied
1^st^ ray cut out	The removal of the mediodistal section (sub 1^st^ ray) of the final orthosis
1^st^ MTPJ	The removal of the mediodistal section (sub 1^st^ MTPJ) of the final orthosis
Plantar fascia groove	A groove that transverses the long axis of the orthosis (sub medial slip of the plantar aponeurosis)
Metatarsal dome	A dome-shaped pad applied under or slightly proximal to the plantar aspect of the metatarsal heads
Intrinsic	The dome is removed from the plaster of the positive cast
Extrinsic	An external dome shaped pad is applied to the final orthosis
Cuboid filler	A increased plaster expansion applied to sub-calcaneocuboid area of the positive cast
Heel aperture	A circular area removed from the plantar heel cup of the shell of the final orthosis

Notes: MTPJ = metatarsophalangeal joint. Negative cast - plaster cast impression of the foot (generally made from plaster or paris bandage), Positive cast - plaster mould of the foot that is formed as the result of liquefied plaster poured into the negative cast.

The rearfoot section covered pouring and balancing of the negative cast, use of a rearfoot post and a medial heel (Kirby) skive [30]. The midfoot section covered the amount of arch fill plus the use of a medial and lateral flange/flare. The forefoot section covered the use of a forefoot post only. Lastly, the accommodations and materials section covered cast and orthotic accommodations and choice of orthotic material. Participants were asked to estimate their percentage use of these prescription variables and detail when, if ever, each variable would be prescribed for adults with symptomatic flexible pes planus. A final summary question listed eight commonly associated signs of symptomatic flexible pes planus and asked participants to indicate their preference for which type of customised FO (e.g. modified Root device, or inverted (Blake) device (Additional file 2)) they would most frequently prescribe [18,31,32]. For example, participants were asked to indicate what type of customised FO would be most frequently prescribed in the presence of a moderately lowered navicular height (Additional file 2). In total, round one involved 15 questions relating to 28 individual variables that may be prescribed for adults with symptomatic flexible pes planus and a summary question to determine preference for the type of FO prescribed across eight presentations of flexible pes planus.

**Figure 2:**
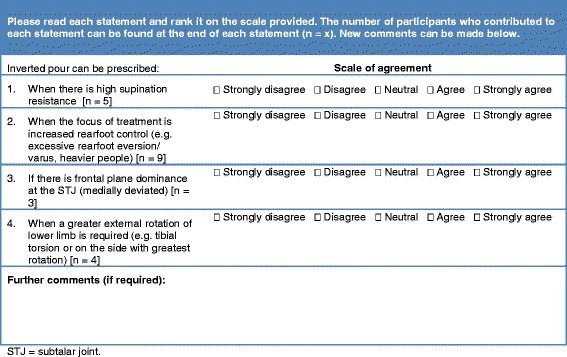
**Round two of the Delphi survey on the use of FOs for flexible pes planus in adults.** Example taken from the rearfoot section, inverted cast pour [heel in an inverted position].

Round two was based on the analysis of responses received in round one (Figure 1). Participants’ comments from round one that related to when, if ever, they would prescribe individual variables were compiled into a list of statements that summarised the full panels’ rationale for prescription. These statements were distributed to participants in round two. Participants were asked to consider each statement, indicate their level of agreement on a five point Likert scale (i.e. strongly disagree, disagree, neutral, agree or strongly agree), and comment further if desired. As an example, statements related to the prescription of an inverted cast pour [where the cast is poured with the heel bisection in an inverted position] for symptomatic flexible pes planus in adults are displayed in Figure 2. Rounds three and four were developed from responses received during previous rounds in the same manner as round two (Figure 1).

### Procedure

Participants were given the following definition of symptomatic flexible pes planus:

*A deformity that involves pain, fatigue (i.e. painful) or gross biomechanical abnormality (i.e. poorly functioning) that would result in your prescription of foot orthoses during the course of your normal practice. Typical clinical indicators of this foot type are excessive motion around the subtalar joint and rearfoot eversion. This group may also include adult- acquired flatfoot (tibialis posterior dysfunction – non-fixed deformity) and involve ligamentous laxity, obesity, joint mal-alignment and muscle contractures.*

When considering questions and statements, participants were also asked to accept that:

*The feet were symptomatic (painful or non-functioning); the decision had been made to prescribe custom(ised) foot orthoses; no other pathology existed to influence the prescription choices;* and *it was limited to the adult client*.

Participants were given two weeks to respond to each survey round. Late responders were sent a reminder with a further two weeks extension. Participants were considered non-responders if they failed to complete the survey within one month of the distribution date and had not requested extra time.

### Data analysis and management

The outcomes of interest were *consensus* and *agreement* on the use of individual prescription variables and the type of customised FO used for symptomatic flexible pes planus in adults. Consensus was based on existing prescription habits and agreement was based on the rationale for the existing prescription habits.

Consensus was determined from round one responses only. It was assessed by: (i) the percentage use of individual prescription variables (Table 2), and (ii) the preference for the type of FO that was used for the eight listed presentations of flexible pes planus (Table 3). Consensus was pre-determined at 70%, so for consensus to be accepted the full panel were required to report their combined estimated use or preference as 70% or more for an individual prescription variable or type of FO respectively. Percentage data for estimated use of individual prescription variables were assessed for normality and found to be skewed; therefore, reported findings are based on group medians (Table 2). Calculation of percentage for the type of FO was based on the number of participants that indicated their preference for each tabled customised orthosis, relative to the number of respondents (Table 3).

**Table 2:** **Participants estimated use of individual prescription variables for symptomatic flexible pes planus in the adult (outcomes reaching consensus are bolded)**

**Section**	**Prescription variable**	**Median%**	**SD**	**Range**
Rearfoot	Cast Pour			
	Neutral [heel in a vertical position]	60.0	32.4	0 - 100
	Inverted [heel in an inverted position]	50.0	31.9	0 - 90
	Everted [heel in an everted position]	0.0	7.2	0 - 30
	Medial heel (Kirby) skive	25.0	29.9	0 - 100
	Post			
	No post	10.0	25.1	0 - 100
	Extrinsic	50.0	32.7	0 - 100
	Extrinsic (inverted)	20.0	28.2	0 - 90
	Extrinsic (everted)	0.0	4.4	0 - 10
	Extrinsic (with motion)	0.0	28.4	0 - 100
Midfoot	Arch fill			
	Minimal	40.0	35.8	0 - 100
	Standard	50.0	28.5	0 - 90
	Maximum	5.0	16.3	0 - 50
	Flange			
	Medial	10.0	28.9	0 - 100
	Lateral	0.0	7.2	0 - 30
Forefoot	Post			
	No post	22.3	28.4	0 - 90
	Intrinsic	43.6	39.2	0 - 100
	Extrinsic	11.0	11.6	0 - 50
	Balanced to perpendicular	50.4	40.8	0 - 100
Accommodations and materials	1^st^ ray cut out	13.1	16.5	0 - 60
	1^st^ MTPJ cut out	15.3	21.0	0 - 100
	Plantar fascia groove	25.0	31.4	0 - 100
	Metatarsal dome			
	Intrinsic	5.0	28.6	0 - 100
	Extrinsic	20.0	21.3	0 - 80
	Cuboid filler	10.0	28.7	0 - 100
	Heel aperture	0.0	14.9	0 - 50
	Polyolyenes (e.g. polypropylene)	**75.0**	31.3	0 - 100
	Cellular foam (e.g. EVA)	10.0	18.2	0 - 70
	Composite (e.g. carbon graphite)	10.0	27.1	0 - 98
	Other	0.0	4.2	0 - 20

Notes: MTPJ = metatarsophalangeal joint, EVA = Ethylene-vinyl acetate.

**Table 3:** **Percentage of respondents that indicated preference for the type of customised FO prescribed for each listed presentation of symptomatic flexible pes planus in the adult (outcomes reaching consensus are bolded)**

**Please indicate your preferred FOs for the listed presentations of symptomatic flexible pes planus in adults (mean%)**	**Modified root device (poured to neutral)**			**Modified root device (poured to inverted)**			**Inverted (Blake or DC wedge) device**			**Forefoot posts**	
	**alone**	**+ skive**	**Total**	**alone**	**+ skive**	**Total**	**alone**	**+ skive**	**Total**	Balanced*	**Other**
Moderate rearfoot eversion	37	11	48	26	7	34	11	4	15	**90**	10
Considerable rearfoot eversion	4	18	22	18	14	32	25	14	39	**89**	11
Moderate talonavicular bulging	21	14	35	21	14	35	7	7	14	**100**	0
Considerable talonavicular bulging	14	18	32	4	11	15	25	11	36	**100**	0
Moderately lowered navicular position	46	7	53	14	7	21	11	0	11	**100**	0
Considerably lowered navicular position	14	14	28	21	14	35	14	7	21	**100**	0
Rigid forefoot tilt (varus or valgus)	21	18	39	21	4	25	7	4	11	**79**	21
Flexible forefoot tilt (varus or valgus)	33	8	41	17	4	21	17	4	21	**81**	19

Notes: poured to neutral = neutral cast pour [heel in a vertical position], poured to inverted = inverted cast pour [heel in an inverted position], *Balanced = forefoot to be balanced parallel to the supporting surface.

Agreement was sought in rounds two, three and four and also determined by percentage, however this was based on the number of participants who agreed with the rationale of a statement relative to the number of respondents to that statement. So, for the use of an individual prescription variable to be accepted, 70% or more of respondents were required to indicate that they agreed or strongly agreed with the rationale of the statement for the use of that variable (Figure 1). For example, in round one where 100% of participants responded, at least 17 of the 24 respondents (i.e. ≥70%) were required to indicate they agreed or strongly agreed with the statement for it to be accepted. Statements where less than 50% of the respondents agreed were excluded. Statements receiving between 50 to 69% agreement were reviewed in the following round to ensure adequate consideration from the panel (Figure 1).

Comments provided by participants in a previous round were collated, themed and paraphrased into statements through discussion and agreement of all four authors. The resulting statements were provided to the participants in the next round of the survey with their own original responses from the previous round, to ensure they were satisfied with the management of the data, and so they could review their responses in light of the collated responses.

Data obviously not related to the initial prescription of customised FOs for symptomatic flexible pes planus in adults or not reaching 70% consensus or agreement at the end of the four round Delphi study were excluded (Figure 1). An *a priori* decision was made that the Delphi survey would conclude when the response rate dropped below 70%, or round four was complete, irrespective of consensus [29]. Descriptive statistics were undertaken in Microsoft Excel 2010 (Microsoft Corp, Redmond Washington, USA).

## Results

### Participant characteristics

The 24 participants were predominantly male (20 male: 4 female) with an average age of 46.3 years (SD 8.7, range 35 to 67 years). Participants had been practicing for an average of 22.6 years (SD 7.4, range 12 to 39 years) and 16 either held or were currently working towards a recognised post-graduate qualification. Two thirds of the participants listed more than one employment setting; 79% identified their primary position as a clinician and 91% worked in a clinical setting at least in a part-time capacity (Additional file 3).

### Survey findings

#### Consensus

Two prescription variables reached consensus in round one. Firstly, polyolefin (e.g. polypropylene) was estimated by the participants to be the most frequently prescribed shell material (75% consensus) (Table 3). Secondly, for the eight listed presentations of flexible pes planus, prescription of a forefoot post that was balanced to perpendicular (to the rearfoot) was the most frequently prescribed forefoot option (79 to 100% consensus) (Table 3). No further consensus was reached.

However, there were several other variables that were frequently prescribed. For the rearfoot, a neutral cast pour [where the cast is poured with the heel bisection in a vertical position] (median use 60%), and an extrinsic rearfoot post (median use 50%), were frequently prescribed variables (Table 3). For the midfoot, a standard arch fill (median use 50%) was the most frequently prescribed variable (Table 2). For the forefoot and accommodation sections, a forefoot post that balanced the forefoot to perpendicular (median use 50%) and a plantar fascia groove (median use 25%) were the most frequently prescribed variables respectively (Table 2). In addition, a modified Root-style device (poured to neutral [heel in a vertical position]), with or without a medial heel (Kirby) skive, was the most frequently prescribed type of FO in the presence of moderate rearfoot eversion (48%), a moderately lowered navicular position (53%), a rigid forefoot varus or valgus (39%), or a flexible forefoot varus or valgus (41%) (Table 3). Both this type of FO and a modified Root-style device (poured inverted [heel in an inverted position]), with or without a medial heel (Kirby) skive, were equally estimated to be the most frequently prescribed in the presence of a moderate talonavicular bulge (35% respectively). A modified Root-style device (poured inverted [heel in an inverted position]) was also estimated to be the most frequently prescribed in the presence of a considerably lowered navicular position (35%). An inverted (Blake) style device, with or without a medial heel (Kirby) skive, was the most frequently prescribed in the presence of considerable rearfoot eversion or considerable talonavicular bulge (39 and 36% respectively) (Table 3).

#### Agreement

Round one resulted in the development of 164 statements to be considered in round two (Table 4). After round two, 24 statements were accepted, 98 statements were excluded and 42 statements required review. In addition, 21 new comments were developed in 20 statements to be considered in round three (Table 4). After round three, 25 statements were accepted, 33 statements were excluded and 4 statements required review (Table 4). No new comments were received in round three. After round four, three statements were accepted and one statement excluded (Table 4).

**Table 4:** **Results per round for statements of agreement considered by participants**

**Round**	**Response rate%**	**Statements accepted (n)**	**Statements not accepted (n)**	**Statements generated (n)**
1	100	NA	NA	164
2	100	24	98	20
3	91	25	33	0
4	91	3	1	NA

Notes: NA = not applicable.

For the rearfoot section, 18 of the 66 statements were accepted (Table 5). Participants agreed that a neutral cast pour [heel in a vertical position] may be prescribed if it reflects the foot position and adequately addresses rearfoot control (Table 5). However, an inverted cast pour [heel in an inverted position], the addition of a medial heel (Kirby) skive, or an extrinsic rearfoot post (inverted) can be prescribed if there is a medially deviated subtalar joint axis [33,34], an increased requirement for rearfoot control, tibialis posterior dysfunction (adult-acquired flatfoot) or a high supination resistance [35,36] (Table 5). There was also agreement from the participants that a medial heel (Kirby) skive may be prescribed when there are concerns about device ‘bulk’ or when greater anti-pronation force is required (Table 5). Furthermore, participants agreed that stability of the device is maximised by an extrinsic rearfoot post, whereas no additional rearfoot post may be prescribed when footwear accommodation is a concern (Table 5). There was no agreement on clinical indicators for the prescription of an extrinsic rearfoot post (everted) or a rearfoot post with motion (Additional file 4).

**Table 5:** **All accepted statements from the Delphi survey on prescription of customised FOs for symptomatic flexible pes planus in the adult**

**In the prescription of FOs for symptomatic pes planus, the following can be prescribed when…**		**Agreement (%)**	**Round accepted**
Inverted cast pour [heel in an inverted position]	The focus of treatment is increased rearfoot control (e.g. excessive rearfoot eversion/varus)	88.3	2
	The STJ is medially deviated (frontal plane dominance)	70.8	2
	There is tibialis posterior dysfunction	79.1	2
	There is a high supination resistance	81.8	3
Neutral cast pour [heel in a vertical position]	To reflect the foot position accurately	75.0	2
	When rearfoot control is adequately addressed in this position	91.7	2
	When inversion cannot be tolerated	81.8	3
Medial heel (Kirby) skive	When there is medial deviation of the STJ axis	91.6	2
	When additional rearfoot control required	100.0	2
	With tibialis posterior dysfunction	75.0	2
	With high supination resistance	86.3	3
	Allows increased control without bulk	86.4	3
	Greater anti-pronation force required at sustentaculum tali	86.4	3
	To increase the calcaneal inclination in the sagittal plane as well as some inversion in frontal plane	81.8	3
No rearfoot post	When footwear accommodation is a concern	75.0	2
Extrinsic rearfoot post	To increase stability of device	91.6	2
Extrinsic rearfoot post (inverted)	To increase rearfoot control (medially deviated STJ, high supination resistance)	79.2	2
	In tibialis posterior dysfunction	90.9	3
Minimal arch fill	To ensure foot posture captured is appropriately maintained	75.0	2
	To achieve full amount of correction (when foot ROM allows)	79.1	2
Maximum arch fill	In the presence of range of motion limitations	72.7	3
	When there is a severe flat foot deformity (e.g. weight bearing medial cuneiform)	86.4	3
Medial flange	In the presence of large midfoot transverse ROM (talus and/or navicular)	82.6	2
	With tibialis posterior dysfunction	75.0	2
	When increased medial control is required (midfoot support)	81.8	3
No forefoot post	In the presence of forefoot supinatus when the supinatus can be reduced	72.7	4
Intrinsic forefoot post	In the presence of forefoot valgus	70.8	2
	To balance forefoot to rearfoot misalignment	79.2	2
	When the inverted rearfoot position offers sufficient support to the symptomatic pes planus foot	72.7	3
	With severe forefoot supinatus or osseous varus	90.9	3
Extrinsic forefoot post	In severe midfoot collapse or fixed inverted forefoot deformities	77.3	3
Forefoot post balanced to perpendicular	Standard practice outside of fixed forefoot deformities	75.0	2
	To encourage the forefoot to be parallel with the supporting surface (offers stability)	81.8	3
	Maintains rearfoot to forefoot balance	95.4	3
1^st^ MTPJ cut out	In the presence of a plantar flexed 1^st^ ray	72.7	3
Plantar fascial groove	When the plantar fascia is tight	83.3	2
	When the plantar fascia is prominent (bowstrings)	91.7	2
	When the plantar fascia is irritated or painful	100.0	3
	To minimise risk of irritation	81.8	4
Metatarsal dome	When forefoot pain exists (e.g. neuroma, bursitis, hyperkeratosis, metatarsalgia)	87.5	2
	In the presence of digital deformities (claw/hammer toes)	83.4	2
	If previously had success with a metatarsal dome	81.8	3
Cuboid filler	Symptomatic lateral column or midfoot (e.g. subluxed cuboid)	90.9	3
Heel aperture	In the presence of plantar calcaneal bursitis	79.0	4
A rigid, semi-rigid and flexible device	Patient weight/size (increased weight = increased rigidity required)	91.7	2
	Degree of control required (increased control = increased rigidity required)	87.5	2
	Activity levels (increased activity = increased rigidity required)	77.3	3
	Perceived tolerance of patient to rigidity	90.9	3
	Footwear limitations	72.7	3
	Available ROM/joint integrity	81.8	3
	Longevity required from device	72.7	3
	Stability is gained with maximum rigidity	72.7	3

Notes: STJ = subtalar joint, ROM = range of motion, MTPJ = metatarsophalangeal joint.

For the midfoot section, 7 of the 34 statements were accepted (Table 5). The participants agreed a minimum arch fill ensures that the captured foot posture is maintained, which achieves full support (or ‘correction’). In contrast, a maximum arch fill may be prescribed in the presence of range of motion limitations or severe flatfoot deformity (Table 5). A medial flange may be prescribed if there is a large amount of transverse plane midfoot motion, tibialis posterior dysfunction (adult-acquired flatfoot) or if increased medial midfoot control is required (Table 5). There was no agreement on clinical indicators for the prescription of a standard arch fill or a lateral flange (Additional file 4).

For the forefoot section, 9 of the 25 statements were accepted (Table 5). Participants agreed that, no forefoot post may be prescribed in the presence of a reducible forefoot supinatus, a soft tissue or positional varus of the forefoot relative to the rearfoot [18], whereas an extrinsic forefoot post may be prescribed in the presence of severe midfoot collapse or a fixed inverted forefoot deformity (Table 5). An intrinsic forefoot post may be prescribed for a severe forefoot supinatus or (osseous) varus, or when the foot exhibits a forefoot valgus, a forefoot to rearfoot misalignment that can be balanced, or when inverting the rearfoot position offers sufficient support (Table 5). However, participants also agreed that outside of fixed forefoot deformities (e.g. reducible forefoot supinatus), balancing the forefoot to perpendicular is considered standard practice as it maintains the rearfoot to forefoot balance and encourages the forefoot to be parallel with the supporting surface (Table 5).

For the accommodations and materials section, 18 of 59 statements were accepted (Table 5). Participants agreed that the use of a 1^st^ metatarsophalangeal joint (MTPJ) cut out may be used in the presence of a plantar-flexed 1^st^ ray and a metatarsal dome may be prescribed for forefoot pain (e.g. sub-metatarsal pain), digital deformities (e.g. flexible clawed toes) or if a metatarsal dome has been used with success previously (Table 5). A plantar fascial groove may be prescribed for a tight, prominent or symptomatic plantar fascia and a cuboid filler prescribed for a symptomatic lateral column or midfoot (Table 5). Participants also agreed that a heel aperture may assist with plantar calcaneal bursitis (Table 5). In relation to materials, a more rigid material can be used when a person is heavier, has increased activity levels, has a foot that requires increased support (i.e. ‘control’), or if the FO needs to be more stable or longer lasting. In contrast, reduced thickness and rigidity of the shell material may be chosen if there are tolerance issues, footwear limitations or reduced joint range of motion (Table 5). There was no agreement on clinical indicators that would result in the prescription of a 1^st^ ray cut out (Additional file 4).

### Development of results into consensus-based recommendations

All included data were pooled and delineated into four sections; rearfoot, midfoot, forefoot, and accommodations and materials. The consensus point that polyolefin (e.g. polypropylene) be used as a base material was considered an overall recommendation (http://www.itek.com.au/news-resources/publications/item/footprop.html). However, the second consensus point, that a forefoot post be balanced to perpendicular was considered in light of two conflicting accepted statements. The first of these was that no forefoot post may be prescribed in the presence of forefoot supinatus if the supinatus can be reduced (i.e. there is sufficient motion available for it to function parallel with the supporting surface during weightbearing) (Table 5). The second was that an extrinsic forefoot post could be prescribed when there was a fixed inverted forefoot deformity or severe midfoot collapse (Table 5). Therefore, the recommendation was developed that; other than a reducible forefoot supinatus, a fixed inverted forefoot deformity or severe midfoot collapse, the forefoot post should be intrinsic and balanced to perpendicular to the rearfoot (http://www.itek.com.au/news-resources/publications/item/footprop.html).

It was also noted that all the accepted statements on the prescription of an inverted cast pour [heel in an inverted position] were consistent with those accepted for the use of a medial heel (Kirby) skive (Table 5). That is, it was accepted that both variables may be prescribed when increased rearfoot control is required or in the presence of a medially deviated subtalar joint axis, tibialis posterior dysfunction (adult-acquired flatfoot) or a high supination resistance (Table 5). This suggests that these two prescription variables are interchangeable, based on practitioner preference or determined by means not captured by this study. Given that both variables reached the inclusion criteria and no evidence exists to guide the choice of one variable over another, the recommendations include both prescription variables with the decision to be at the discretion of the practitioner (http://www.itek.com.au/news-resources/publications/item/footprop.html).

Following analysis of the findings from this Delphi survey, the consensus variables and the accepted statements were developed into a proforma, the consensus-based *Foot orthosis Prescription Recommendations for symptOmatic flexible Pes planus in adults (FootPROP)*, which can be modified to co-exist with current foot orthosis prescription forms (http://www.itek.com.au/news-resources/publications/item/footprop.html).

## Discussion

This study has resulted in the first consensus-based recommendations for the prescription of customised FOs for symptomatic flexible pes planus in adults. Both clinicians and researchers may use these recommendations to guide their practice and research. Although the findings of this study suggest that existing prescription habits of expert podiatrists for this condition are not universal, they do indicate that there is agreement on the rationale for use of individual prescription variables (Table 5).

The only previous study relating to practitioner prescribing of foot orthoses by Landorf et al. surveyed Australian and New Zealand podiatrists on their prescription habits in 2001 [37]. They reported that the estimated use of a modified Root-style device was just over half (52%) of customised FO prescriptions. The findings from our study confirm that the prescription variables common to a standard modified Root-style device are still the most frequently prescribed; that is, a neutral cast pour [heel in a vertical position], a standard arch fill and a forefoot post balanced to perpendicular (Table 2). The standard modified Root-style device was also the most frequently reported preference for five of the eight listed presentations of symptomatic flexible pes planus in adults (4 to 46%) (Table 3). However, these results demonstrate that a standard modified Root-style device is not universally prescribed for symptomatic flexible pes planus in adults, with variations based on several identified rationales. From participants’ responses, the aim of prescription for this population is to: (i) influence the level of ‘control’ of the rearfoot and midfoot with cast pour, rearfoot post, medial heel (Kirby) skive, arch fill and rigidity of the shell choices, (ii) influence or support the forefoot position with forefoot post choices, and (iii) accommodate or minimise painful pathologies and deformities with the accommodation choices.

The findings of our study show that when ‘increased control’ of the rearfoot is required, prescription of an inverted cast pour [heel in an inverted position], a medial heel (Kirby) skive or an extrinsic rearfoot post (inverted) may be employed (Table 5). The rationale for these prescription options have been previously discussed in the literature [18,30,38-41]. The findings of our study suggest that these prescription variables may be employed interchangeably or as adjunctive options, with the use of an inverted cast pour [heel in an inverted position], specifically, estimated to be prescribed in 50% of symptomatic flexible pes planus presentations (Table 2).

For the midfoot, ‘increased control’ may be achieved with the prescription of a minimal arch fill and/or the use of a medial flange (Table 5). Both prescription variables are purported to reduce excessive pronation and assist re-supination of the foot during the mid-stance and propulsive (terminal) phases of gait [40], although there is currently no evidence to support this. The participants in our study estimated that 40% of prescriptions used a minimal arch fill, again making this a frequently prescribed variation on a ‘standard’ FO (Table 2).

For the forefoot, the rationale for accepted prescription variables related to the existing forefoot to rearfoot alignment and was dependent on available forefoot motion (Table 5). For example, if there was a reducible forefoot supinatus, it was accepted that balancing the forefoot to perpendicular encourages forefoot alignment to be parallel to the supporting surface (Table 5). However, in the presence of forefoot motion limitations, the forefoot may be targeted for support with an extrinsic forefoot post (Table 5), or held in its existing alignment by the prescription of no forefoot post (Table 5). This is in keeping with the literature, which suggests that the aim of extrinsic forefoot posting is to minimise the compensatory effects of a large non-reducible forefoot misalignment [42], whereas when the existing forefoot position is perpendicular to the rearfoot in the frontal plane, capturing this existing ‘aligned’ position offers sufficient support.

For accommodations, the rationale for prescription appeared primarily based on the presence of existing pathologies (Table 5). Participants agreed that the aim was to accommodate painful conditions (e.g. the use of a plantar fascial groove in the presence of an irritated or painful plantar fascia) or to offload painful plantar metatarsal head regions (e.g. with a metatarsal dome) (Table 5). The recommendations for the use of a semi-rigid shell material (i.e. polyolefin), appeared targeted at ensuring the FO offers sufficient control of the flexible foot type (Table 5), with the choice of final ‘rigidity’ guided by perceived client tolerance, footwear concerns and joint integrity or range of motion limitations (Table 5).

Alternative published prescription recommendations for flexible pes planus in adults are sparse. Nonetheless, some similarities exist between the available literature and the outcomes reported in our study. For example, Scherer suggested that in the presence of tibialis posterior dysfunction (adult-acquired flatfoot), a neutral cast pour [heel in a vertical position] with the addition of a medial flange and a medial heel (Kirby) skive be prescribed [23], which is consistent with our findings. Interestingly, Scherer does not advocate specific prescription recommendations for other presentations of flexible pes planus in the adult, only for tibialis posterior dysfunction. Similarly, Rosenbloom recommended a neutral cast pour [heel in a vertical position] with a medial flange, but also recommended an extrinsic rearfoot and forefoot post, with this recommendation aimed at all presentations of flexible pes planus [24]. The findings of our Delphi survey have established that standardising the prescription for flexible pes planus presentations may not reflect existing clinical practice in Australia. Within the limits of this study, this supports the perception that standardising the FOs used in research may not mirror current clinical practice. It is important to note, however, that this issue relates to generalisability of the orthotic prescription, and the authors cannot make any conclusions as to whether this influences the effectiveness of FOs used in either setting. There is no evidence to suggest that one approach to FOs prescription is more, or less, effective than another.

The need to investigate the effectiveness of individualised FOs for adults with symptomatic pes planus is clear. The development of the FootPROP proforma will allow researchers to prescribe customised FOs that are specific for individual signs and symptoms of each study participant. It also offers clinicians the opportunity to review consensus-based recommendations in relation to their own practice methods. The FootPROP proforma requires further evaluation, however it is the first consensus-based prescription recommendations for this specific population and it is envisaged that it will allow for more generalisable research outcomes in the future.

Our findings need to be viewed in the context of several limitations. Firstly, there is no universally accepted definition or classification for flexible pes planus, or consensus on what or when intervention may be required. Secondly, although our findings are a good starting point, they are based on expert opinion, which in the context of evidence-based practice constitutes low-level evidence. In particular, the findings outlined in this study do not constitute evidence that the prescription recommendations lead to an effective FO. Further evaluation with studies that provide higher level evidence (e.g. randomised trials) are needed. Thirdly, the Delphi survey process has been subject to concerns, with the existence of consensus or agreement not necessarily confirming that the answer is correct, as there is “*… a danger of deriving collective ignorance rather than wisdom”* [26]. Furthermore, anonymity and confidentiality are suggested requirements of participants in Delphi surveys as collusion may affect results. However, given the general collegiate relationships that exist within the Australian podiatric profession, it cannot be guaranteed that participants did not deduce who their fellow survey participants were. Finally, the term ‘expert’ and its application to health practitioners is controversial [27] and no classification exists within the podiatry profession for ‘expert status’. The criteria set for expert within this study were based on discussions between authors and criteria set in other Delphi panels. Accordingly, the findings of this study may not be generalisable to the wider population of podiatric practitioners. This issue extends to podiatrists from other countries, where differences in prescription habits for FOs for adults with flexible pes planus may exist.

## Conclusion

This study found some consistency in FO prescription habits for symptomatic flexible pes planus in adults and that the prescription of individual variables for this condition is based on the following four key rationales. Firstly, when the required foot position is adequately supported in a neutral position, then a standardised modified Root style device (i.e. a neutral cast pour [heel in a vertical position], a standard arch fill and a forefoot post that is balanced to perpendicular) may offer adequate control and support. However, if the aim of the intervention is to gain ‘increased control’, then the use of an inverted cast pour [heel in an inverted position], an extrinsic rearfoot post (inverted), a medial heel (Kirby) skive, minimal arch fill and/or or a medial flange are modifications that can be incorporated. Secondly, the choice of a forefoot post can be directed by the existing forefoot position. It may be: held in its existing position (no forefoot post), influenced to align the forefoot with the rearfoot when motion allows (an intrinsic balanced forefoot post), or supported in the presence of motion limitation (an extrinsic forefoot post). Thirdly, prescribing orthotic accommodations can be directed by the presence of painful pathologies or digital deformities. Fourthly, the use of polyolefin (e.g. polypropylene) as a base material is recommended, with the final rigidity guided by client characteristics and perceived requirements. Finally, the FootPROP proforma, developed as a result of these findings, will allow investigators evaluating the effectiveness of FOs for flexible pes planus to adopt prescriptions that are more reflective of clinical practice.

## Additional files

Additional file 1:
**Population based calculations for the Delphi survey panel formation.**
Additional file 2:
**Round one for the Delphi survey on the prescription of customised FOs for symptomatic flexible pes planus in adults.**
Additional file 3:
**Current employment classification of participants.**
Additional file 4:
**Results for the Delphi survey on the use of FOs for symptomatic flexible pes planus in the adult; excluded statements (bolded statements reached agreement but were outside of the scope of the study).**
