# Supplementary material for: Consensus-based recommendations of Australian podiatrists for the prescription of foot orthoses for symptomatic flexible pes planus in adults
Source: J Foot Ankle Res. 2014 Nov 25;7:49. doi: 10.1186/s13047-014-0049-2 (PMC4282733; doi:10.1186/s13047-014-0049-2)
Supplement: Additional file 1: — Population based calculations for the Delphi survey panel formation. [file 13047_2014_49_MOESM1_ESM.docx]

**Additional file 1: Population based calculations for the Delphi survey panel formation**

|  | **Australian population (2010)** | **% of Australian population** | **Calculated number of participants relative to population** | **Final number of participants** |
| --- | --- | --- | --- | --- |
| New South Wales | 7317500 | 32.2 | 8.0 | 7 |
| Victoria | 5640900 | 24.9 | 6.2 | 5 |
| Queensland | 4599400 | 20.3 | 5.2 | 4 |
| South Australia | 1659800 | 7.3 | 1.8 | 2 |
| Western Australia | 2366900 | 10.4 | 2.6 | 3 |
| Tasmania | 511000 | 2.3 | 0.6 | 1 |
| Northern Territory | 231200 | 1.0 | 0.3* | 1 |
| Australian Capital Territory | 366900 | 1.6 | 0.4* | 1 |
| Total | 22693600 | 100.0 | 25.0 | 24 |

Notes: *rounded up to a full allocation.
