# Supplementary material for: Consensus-based recommendations of Australian podiatrists for the prescription of foot orthoses for symptomatic flexible pes planus in adults
Source: J Foot Ankle Res. 2014 Nov 25;7:49. doi: 10.1186/s13047-014-0049-2 (PMC4282733; doi:10.1186/s13047-014-0049-2)
Supplement: Additional file 2: — Round one for the Delphi survey on the prescription of customised FOs for symptomatic flexible pes planus in adults. [file 13047_2014_49_MOESM2_ESM.docx]

**Additional file 2: Round one for the Delphi survey on the prescription of customised FOs for symptomatic flexible pes planus in adults.**

**The Delphi survey: round one.**

**Instructions to participants;**

Pes planus can be multifactorial in both origin and presentation however our prescription options for foot orthoses (FO) are somewhat limited to influencing the rearfoot, midfoot or forefoot position and function. This survey is aimed at determining when and how expert podiatrists approach the individual prescription variables to gain best influence over the position and function when it comes to pes planus. The survey itself is divided into four sections: rearfoot, midfoot, forefoot and material choice and accommodations.

Our choice of 'device type' has traditionally also been limited to a modified Root device (foot captured in neutral with the forefoot balanced to perpendicular) or an 'inverted' device (i.e. Blake or DC wedge) with the optional addition of a medial heel (i.e. Kirby) skive and other accommodations. This survey is based on the assumption that you prescribe (or would prescribe) one of the above mentioned 'types' of devices - If you vary from this please indicate how and when in the relevant comments fields below.

In the majority of questions below you are provided with a scale of 0% to 100%. We ask that you indicate along this scale the position that best reflects your orthotic prescription habits for each variable with 0% meaning you never prescribe this variable and 100% meaning you always prescribe this variable. Following this we ask that you explain your prescription choices with the emphasis on what influences your decisions for each variable. For the sake of consistency we have used traditional manufacturing terminology throughout this survey.

We remind you that this is in regard to adult clients only and that pes planus, by our definition, has a level of rearfoot eversion involved.

| **What best reflects your prescription habits for the following for the adult with symptomatic flexible pes planus…** | | |
| --- | --- | --- |
| **Variable** | **% of use** | **Detail when, if ever, you would choose this option and why** |
| **Rearfoot** | | |
| Cast Pour; |  |  |
| - *Inverted* |  |  |
| - *Neutral* |  |  |
| - *Everted* |  |  |
| Medial heel (Kirby) skive |  |  |
| Rearfoot post (heel stabiliser); |  |  |
| - *No post* |  |  |
| - *Extrinsic (inverted)* |  |  |
| - *Extrinsic (neutral)* |  |  |
| - *Extrinsic (everted)* |  |  |
| - *Extrinsic (with motion)* |  |  |
| **Midfoot** | | |
| Arch fill: |  |  |
| - *Minimal* |  |  |
| - *Standard* |  |  |
| - *Maximum* |  |  |
| Flange: |  |  |
| - *Medial* |  |  |
| - *Lateral* |  |  |
| **Forefoot** | | |
| Forefoot post: |  |  |
| - *No post* |  |  |
| - *Intrinsic* |  |  |
| - *Extrinsic* |  |  |
| - *Balanced to perpendicular* |  |  |
| **Accommodations and materials** | | |
| 1^st^ ray cut out |  |  |
| 1^st^ metatarsal cut out |  |  |
| Plantar fascia groove |  |  |
| Metatarsal dome (intrinsic) |  |  |
| Metatarsal dome (extrinsic) |  |  |
| Cuboid filler |  |  |
| Heel aperture |  |  |
| Polyolyenes (e.g. polypropylene) |  |  |
| Cellular foam (e.g. EVA) |  |  |
| Composite (e.g. carbon graphite) |  |  |
| Other |  |  |

| **Summary section** | | | | | | | | | | | |
| --- | --- | --- | --- | --- | --- | --- | --- | --- | --- | --- | --- |
| Please indicate below (tick) your preference of type of FOs for each presentation of symptomatic flexible pes planus in the adult. | **Modified Root device (poured to neutral)** | | | **Modified Root device (poured to other)** | | | **Inverted (Blake or DC wedge) device** | | | **Forefoot**  **post** | |
|  | **Alone** | **+ skive** | **Total** | **Alone** | **+ skive** | **Total** | **Alone** | **+ skive** | **Total** | **Balanced** | **Other** |
| Moderate rearfoot eversion |  |  |  |  |  |  |  |  |  |  |  |
| Considerable rearfoot eversion |  |  |  |  |  |  |  |  |  |  |  |
| Moderate talonavicular bulging |  |  |  |  |  |  |  |  |  |  |  |
| Considerable talonavicular bulging |  |  |  |  |  |  |  |  |  |  |  |
| Moderately lowered navicular position |  |  |  |  |  |  |  |  |  |  |  |
| Considerably lowered navicular position |  |  |  |  |  |  |  |  |  |  |  |
| Rigid forefoot tilt (varus or valgus) |  |  |  |  |  |  |  |  |  |  |  |
| Flexible forefoot tilt (varus or valgus) |  |  |  |  |  |  |  |  |  |  |  |
