# Supplementary material for: Consensus-based recommendations of Australian podiatrists for the prescription of foot orthoses for symptomatic flexible pes planus in adults
Source: J Foot Ankle Res. 2014 Nov 25;7:49. doi: 10.1186/s13047-014-0049-2 (PMC4282733; doi:10.1186/s13047-014-0049-2)
Supplement: Additional file 3: — Current employment classification of participants. [file 13047_2014_49_MOESM3_ESM.docx]

**Additional file 3: Current employment classification of participants.**

|  | **Academic** | **Clinician** | **Researcher** |
| --- | --- | --- | --- |
| Primary position | 4 | 19 | 1 |
| Second position | 4 | 3 | 3 |
| Third position | 1 |  |  |
