# Supplementary material for: Consensus-based recommendations of Australian podiatrists for the prescription of foot orthoses for symptomatic flexible pes planus in adults
Source: J Foot Ankle Res. 2014 Nov 25;7:49. doi: 10.1186/s13047-014-0049-2 (PMC4282733; doi:10.1186/s13047-014-0049-2)
Supplement: Additional file 4: — Results for the Delphi survey on the use of FOs for symptomatic flexible pes planus in the adult; excluded statements (bolded statements reached agreement but were outside of the scope of the study). [file 13047_2014_49_MOESM4_ESM.docx]

**Additional file 4: Results for the Delphi survey on the use of FOs for symptomatic flexible pes planus in the adult; excluded statements (bolded statements reached agreement but were outside of the scope of the study).**

| **In the prescription of FOs for symptomatic flexible pes planus the following may be prescribed when.. (number of participants who contributed to the statement can be found at the end [n = x]** | **Agreement %** |
| --- | --- |
| **1. Inverted pour** |  |
| *The person is heavier/larger [n = 1]* | 37.5 |
| *In the absence of knee or back pathology [n = 1]* | 16.7 |
| *This approach shifts control to dorsiflexing the 1st metatarsal and potentially destabilising the midfoot [n = 1]* | 12.5 |
| *The pour position does not reflect anything to do with the correction and thus the orthotic outcome [n = 1]* | 4.2 |
| *Greater external rotation of the lower limb is required [n = 4]* | 59.1 |
| **2. Neutral pour** |  |
| *When motion control is required more distally [n = 3]* | 45.8 |
| *Reduce forefoot supinatus prior to pour [n = 1]** | 45.5 |
| **3. Everted pour** |  |
| *When a rigid or fixed deformity exists and neutral cannot be achieved [n = 9]^#^* | **86.4** |
| *With acute or chronic trauma or tendinopathy [n = 2]* | 16.7 |
| **4. Medial heel (i.e. Kirby) skive** |  |
| *If medial plantar nerve irritation is present (remove when settled) [n = 1]* | 8.3 |
| *With moderate supination resistance force with more proximal symptoms [n = 2]* | 37.5 |
| *When unilateral variations are required [n = 1]* | 33.3 |
| *With increased transverse plane motion at rearfoot [n = 1]* | 33.3 |
| *Where there is a large amount of soft tissue spread at calcaneus.[n = 1]* | 8.3 |
| *Where there is rearfoot varus [n = 1]* | 25.0 |
| *When manual supination resistance test is greater than +4 [n = 1]* | 33.3 |
| *In the absence of heel pain [n = 1]* | 33.3 |
| *In the absence of degraded plantar fat pad [n = 1]* | 29.2 |
| *Prefer an inverted device with low correction [n = 1]* | 29.2 |
| *Prefer a hybrid of DC wedge and Blake inverted device [n = 1]* | 8.3 |
| *Skives in rigid devices can create fat pad or medial nerve impingement. Accurate control in device with calculated motion in rearfoot post can avoid this [n = 1]** | 27.2 |
| **5. No rearfoot posts** |  |
| *When there is mild/reduced rearfoot pronation and calcaneal eversion [n = 3]* | 41.7 |
| *When chronic heel pain/plantar fasciitis is present [n = 2]* | 16.7 |
| *To enhance the inverted positioning of the foot [n = 1]* | 0.0 |
| *When there is combined lateral instability [n = 1]* | 12.5 |
| *Only for dance or heel shoes, not everyday shoes. In not a fixed condition should use rearfoot post with motion as calculated [n = 1]** | 18.2 |
| **6. Extrinsic rearfoot posts (neutral)** |  |
| *When there is a joint motion restriction (STJ) [n = 3]* | 41.7 |
| *When varus or valgus wedging has not been tolerated [n = 1]* | 41.7 |
| *When symptoms are more distal (midfoot/forefoot) [n = 1]* | 29.2 |
| *When shoe fit is a concern [n = 1]* | 12.5 |
| *When device is supplying adequate rearfoot control [n = 2]* | 63.6 |
| **7. Extrinsic rearfoot posts (inverted)** |  |
| *As an addition to current device if increased correction required [n = 3]^#^* | **87.5** |
| *When in combination with extrinsic forefoot posts (e.g. forefoot supinatus) [n = 1]* | 37.5 |
| *When NCSP is inverted [n = 1]* | 41.7 |
| *Routinely used unless varus posting not tolerated [n = 1]* | 25.0 |
| *When using a DC wedge orthotic [n = 1]* | 16.7 |
| *With more proximal disorders (patellofemoral disorders etc) [n = 1]* | 41.7 |
| **8. Extrinsic rearfoot posts (everted)** |  |
| *With medial OA of knee/genu valgum [n = 2]* | 45.8 |
| *With marked lateral instability [n = 1]* | 45.8 |
| *With fixed deformity [n = 4] ^#^* | 54.2 |
| *Depending on how symptomatic the foot is compared to the knee, if knee pain was greater may consider everting the post [n = 1]** | 36.4 |
| **9. Extrinsic rearfoot posts with motion** |  |
| *As it comes standard with rearfoot posts [n = 1]* | 0.0 |
| *Depending on level of control and footwear (decreases damage from posting) [n = 1]* | 25.0 |
| *To allow for controlled STJ pronation to the degree measured by mechanical/video assessment [n = 1]* | 12.5 |
| *Always in acrylic heel posts [n = 1]* | 20.8 |
| *Occasionally, with high level of tibial varum and inverted heel strike [n = 1]* | 29.2 |
| *Only in acrylic posts, EVA posts develop motion quickly [n = 1]** | 45.4 |
| *Rearfoot posts should always be calculated to enhance device [n = 1]** | 31.8 |
| **MIDFOOT SECTION** |  |
| **10. Minimal arch fill** |  |
| *When patient is of increased weight or higher joint laxity [n = 3]* | 45.8 |
| *Preference is to measure arch height and prescribe to this height [n = 2]* | 33.3 |
| *In the absence of marked navicular depression or arch pain [n = 2]* | 33.3 |
| *When using lower density or flexible materials [n = 1]* | 41.7 |
| *Routinely in younger patients [n = 1]* | 37.5 |
| **11. Standard arch fill** |  |
| *When the plantar fascia is flexible [n = 2]* | 25.0 |
| *When plantar fascia is prominent [n = 1]* | 20.8 |
| *Prefer to prescribe to a specific arch height [n = 1]* | 25.0 |
| *When in conjunction with a medial skive [n = 1]* | 20.8 |
| *As it is well tolerated and offers adequate control [n = 5]* | 63.6 |
| *It is standard practice in adult patients [n = 5]* | 59.1 |
| *When ROM is controlled from this position (low supination resistance) [n = 3]* | 59.1 |
| **12. Maximum arch fill** |  |
| *When devices are accommodative (not functional) [n = 1]* | 36.4 |
| *When there is vulnerability to distal MLA blistering [n = 1]* | 68.2 |
| **13. Medial flange/s** |  |
| *Usually only in paediatric clients [n = 6]* | 12.5 |
| *A medial flare is a comparable option [n = 4]* | 41.7 |
| *When forefoot is abducted [n = 2]* | 41.7 |
| *Where proprioceptive awareness is desired [n = 1]* | 12.5 |
| *(in statement above) it should not be ‘proprioception’ but ‘exterioception’ [n = 1]* ^#^* | 18.2 |
| *Will not be necessary with correct measure and adequate shell and rear post control, it makes shoe fitting difficult [n = 1]** | 18.2 |
| **14. Lateral flange/s** |  |
| *With chronic lateral ankle instability [n = 4]* | 45.8 |
| *To prevent lateral slippage of the foot on the device [n = 3]* | 45.8 |
| *With severe forefoot abduction on rearfoot [n = 3]* | 29.2 |
| *With cuboid syndrome/symptoms [n = 2]* | 16.7 |
| *When patient is involved in ‘side to side’ motion sports [n = 1]* | 12.5 |
| *In the presence of excessive transverse plane motion [n = 1]* | 16.7 |
| *In conjunction with a medial flange to help minimise excessive transverse motion [n = 1]** | 45.5 |
| **FOREFOOT SECTION** |  |
| **15. No forefoot posts** |  |
| *When a forefoot post may inhibit the windlass effect [n = 1]* | 41.7 |
| *In the presence of forefoot supinatus [n = 1]* | 37.5 |
| *Where minimal control is required [n = 1]* | 33.3 |
| *When no rearfoot to forefoot pathology exists [n = 2]* | 68.2 |
| **16. Intrinsic forefoot posts** |  |
| *In combination with a Blake style device [n = 1]* | 29.2 |
| *To allow more direct capture of forefoot anomaly [n = 2]* | 68.2 |
| *If a slight increase in support from the midfoot to forefoot is required [n = 1]* | 63.6 |
| **17. Extrinsic forefoot posts** |  |
| *In forefoot supinatus – to be removed as supinatus reduces [n = 4]* | 37.5 |
| *Only to increase surface area of distal shell (reduce shoe damage/maintain better control) [n = 2]* | 25.0 |
| *When adjustments will be required [n = 1]* | 20.8 |
| *When late stance phase control is required [n = 1]* | 33.3 |
| *Medium to high mileage runners benefit with this post [n = 1]* | 12.5 |
| *When applying forefoot valgus posting for medial knee joint OA [n = 2]* | 59.1 |
| **18. Balancing the forefoot to perpendicular** |  |
| *For shoe accommodation and comfort [n = 1]* | 41.7 |
| *Midfoot or forefoot symptoms [n = 1]* | 45.8 |
| *Where an osseous forefoot valgus or varus exists [n = 1]* | 45.8 |
| **ACCOMMODATIONS AND MATERIALS SECTION** |  |
| **19. 1^st^ ray cut outs** |  |
| *In the presence of Morton’s toe [n = 2]* | 37.5 |
| *When forefoot supinatus present that is reduced in cast [n = 1]* | 25.0 |
| *With sesamoiditis [n = 1]* | 45.8 |
| *In children – encourage normal plantar flexion of 1^st^ ray and avoid supinatus [n = 1]* | 25.0 |
| *Metatarsus primus elevatus [n = 1]* | 25.0 |
| *Over activity of abductor hallucis [n = 1]* | 20.8 |
| *Increase the windlass function in the presence of functional hallux limitus [n = 1]* | 63.6 |
| *With functional hallux limitus [n = 3]* | 59.1 |
| *A 1^st^ ray cut out makes the device unstable [n = 2]** | 40.9 |
| **20. 1^st^ metatarsal cut outs** |  |
| *With functional hallux limitus [n = 3]* | 45.8 |
| *In the presence of sesamoiditis [n = 2]* | 45.8 |
| *It is standard practice [n = 1]* | 4.2 |
| *In children – encourage normal plantarflexion of 1^st^ ray and avoid supinatus [n = 1] ^#^* | 16.7 |
| *In the presence of metatarsus primus elevates [n = 1]* | 8.3 |
| *Over activity of abductor hallucis [n = 1]* | 16.7 |
| *In relation to sesamoiditis, it would depend if it was the tibial or fibula sesamoid [n = 1]** | 18.2 |
| *The preferred method is a reverse Morton’s extension [n = 1]** | 9.1 |
| **21. Metatarsal domes** |  |
| *With ligamentous laxity (of the metatarsal arch) [n = 3]* | 37.5 |
| *In the presence of an elevated 1st ray [n = 2]* | 29.2 |
| *To improve sagittal motion, functional hallux limitus [n = 2]* | 41.7 |
| *When 2-5 forefoot varus exists [n = 1]* | 41.7 |
| *With a long second metatarsal [n = 1]* | 33.3 |
| *In the presence of plantar flexed 1st ray [n = 1]* | 50.0 |
| *Domes should not place metatarsal heads unevenly. Metatarsal arch should be supported along normal contour with the heads maintaining a parallel to the ground [n = 1]** | 40.9 |
| **22. Plantar fascial grooves** |  |
| *In the presence of hallux limitus/rigidus [n = 1]* | 20.8 |
| *To enable an effective windlass mechanism and improve forefoot/1^st^ ray motion [n = 1]* | 59.1 |
| *As plantar fascia motion should always be allowed for [n = 1]** | 27.3 |
| **23. Cuboid fillers** |  |
| *In the presence of a very high lateral arch [n = 2]* | 45.8 |
| *In the presence of fixed deformity [n = 1] ^#^* | 29.2 |
| *To stabilise the foot (i.e. lateral instability) [n = 5]* | 63.6 |
| *With the use of a hybrid DC wedge/Blake device as it assists the foot to sit better in the shoe [n = 1]** | 36.4 |
| **24. Heel apertures** |  |
| *To accommodate footwear limitations [n = 2]* | 37.5 |
| *In the presence of heel pain [n = 8]* | 54.5 |
| *When heel pain is due to atrophy or lack of fibro fatty padding under medial calcaneal tubercle [n = 1]** | 59.1 |
| **25. The decision to use a rigid, semi-rigid or flexible device may be influenced by…** |  |
| *Angle of force of body mass to the foot [n = 1]* | 41.7 |
| *Fat pad condition [n = 1]* | 33.3 |
| *Age (older = less rigidity) [n = 3]* | 37.5 |
| *Shape determines rigidity more than material choice [n = 1]* | 25.0 |
| *Ability to adjust materials post dispense [n = 4]* | 59.1 |
| *Sporting activity/level [n = 2]* | 66.7 |
| *Should differentiate between hardness vs type of material as EVA is used due to ease of modification but hardness can be altered depending on patient likelihood of adverse effects [n = 1]** | 27.3 |

Notes: * statement generated in round two. # out of study scope. STJ = subtalar joint, NCSP = neutral calcaneal stance position, OA = osteoarthritis, EVA = ethylene-vinyl acetate, ROM = range of motion, MLA = medial longitudinal arch.
